# The Food and Health Environment in Junior Secondary Schools in Urban Burkina Faso: A Cross-Sectional Study of Administrators, Food Vendors and Early Adolescents

**DOI:** 10.3390/ijerph182312689

**Published:** 2021-12-01

**Authors:** Joy Mauti, Isabel Mank, Jan-Walter De Neve, Guillaume Alfred Gyengani, Paul-André Somé, Sachin Shinde, Wafaie Fawzi, Till Bärnighausen, Alain Vandormael

**Affiliations:** 1 Faculty of Medicine, Heidelberg Institute of Global Health (HIGH), Heidelberg University Hospital, Heidelberg University, Im Neuenheimer Feld 130.3, 69120 Heidelberg, Germany; joy.mauti@uni-heidelberg.de (J.M.); isabel.mank@uni-heidelberg.de (I.M.); janwalter.deneve@uni-heidelberg.de (J.-W.D.N.); till.baernighausen@uni-heidelberg.de (T.B.); 2 Institut Superieur des Sciences de la Population (ISSP), Blvd Charles de Gaulle, Ouagadougou 03 BP 7118, Burkina Faso; gguillaumealfred@gmail.com (G.A.G.); paulandre.some@gmail.com (P.-A.S.); 3 Department of Global Health and Population, Harvard T.H. Chan School of Public Health, Boston, MA 02115, USA; sshinde@hsph.harvard.edu (S.S.); mina@hsph.harvard.edu (W.F.); 4 Department of Epidemiology, Harvard T.H. Chan School of Public Health, 677 Huntington Avenue, Boston, MA 02115, USA; 5 Department of Nutrition, Harvard T.H. Chan School of Public Health, 677 Huntington Avenue, Boston, MA 02115, USA; 6 Africa Health Research Institute (AHRI), Africa Centre Building, Via R618 to Hlabisa, Somkhele, P.O. Box 198, Mtubatuba 3935, South Africa; 7 Harvard Center for Population and Development Studies, Harvard University, Cambridge, MA 02138, USA

**Keywords:** nutrition, WASH, policy, students, teacher, schools, Burkina Faso, West Africa

## Abstract

School enrolment rates have increased globally, making the school environment a unique setting to promote healthy nutrition and eating outcomes among early adolescents. In this cross-sectional study, we describe the food and health environment of junior secondary schools in Ouagadougou (Burkina Faso, West Africa). We evaluated the food and health environment using three components: (1) the implementation of health-related policies or guidelines in the schools, (2) the provision of health, nutrition and water, sanitation & hygiene (WASH) services in the schools, and (3) the quality of the school food environment, including foods sold by vendors. We used stratified random sampling to recruit 22 junior secondary schools from the five Ouagadougou districts in 2020. Trained fieldworkers collected standardized questionnaire data from 19 school administrators, 18 food vendors, and 1059 in-school adolescents. We report that only 7 out of 19 school administrators were aware of existing health-related policies and guidelines at their school and only 3 schools had a school health and nutrition curriculum in place. The overall provision of health, nutrition and WASH services was low or inadequate. Likely because of the lack of school canteens, 69% of the students bought snacks and unhealthy foods from food vendors. There is a critical need to improve the food and health environment of junior secondary schools in urban Burkina Faso.

## 1. Introduction

Early adolescence is marked by significant physical and cognitive growth with broad implications for health across the life course [1]. A significant percentage of early adolescents in sub-Saharan Africa are affected by the double burden of malnutrition with high levels of undernutrition (underweight, stunting and micronutrient deficiencies) as well as overweight and anemia [2,3,4,5]. Considering that most children and adolescents are at least for some years in school, a combination of interventions at the school level may positively impact their habits, attitudes and preferences with long-term health benefits for adolescents as they age into adulthood [6,7].

Globally, school enrolment rates have increased consistently over the past years with approximately 84% of early adolescents (11 to 14 years) in school [8]. Schools provide a unique setting to implement nutrition-based interventions. They can promote healthy eating behaviors and attitudes, encourage better hygiene practices and health-care seeking behavior, and reduce obesity, chronic diseases and other health-related problems among students [9,10]. While the majority of school-based interventions aim to increase student participation or improve learning outcomes [8], few focus on the school food and health environment in low- and middle income countries (LMICs) [11,12]. The school food and health environment can be defined as the physical, economic, socio-cultural and policy environment that impacts the food and health choices and outcomes at the school level [11,12,13]. It covers the interaction of the external (availability, prices, quality and marketing and regulations) and internal dimensions (accessibility, affordability, convenience and desirability) of food and health products and services [12,14].

In 2006, the World Health Organization (WHO) proposed the Nutrition-Friendly School Initiative (NFSI) to leverage the school environment (i) to effectively address the double burden of malnutrition (including under- and overweight, nutrient deficiencies, food security, hygiene and health surveillance), and (ii) to promote school-based programs to enhance physical activity and health nutrition practices [15,16]. In order to be accredited as a Nutrition-Friendly School, the initiative requires schools to fulfil 26 essential criteria within five core components. Those five core components request: (i) having a written school policy on nutrition, (ii) enhancing awareness and capacity building, (iii) developing a nutrition and health curriculum, (iv) creating a supportive school environment, and (v) providing supportive school nutrition and health services [15]. Since its inception, the NFSI framework has been implemented to support the food and health environments of schools across 18 countries [16], mainly in European regions, but also in our study region, Burkina Faso [16,17].

In Africa, research on the (school) food and health environment and its impact on early adolescents is limited, with the exception of South Africa [11,14,18]. To better understand the food and health environment across schools in Ouagadougou, Burkina Faso, we undertook a cross-sectional study with school administrators, food vendors and in-school adolescents aged 11 to 14 years. Motivated by the WHO NFSI framework, we specifically focus on three components within the school environment: (1) the availability of health-related policies or implemented guidelines, (2) the provision of health, nutrition and water, sanitation and hygiene (WASH) services, and (3) the quality of the school food environment, including foods sold by vendors. We plan to use the study findings to inform policy and improve the food and health environments of early adolescents attending junior secondary schools in urban Ouagadougou.

## 2. Materials and Methods

### 2.1. Study Site and Design

**Figure 1:**
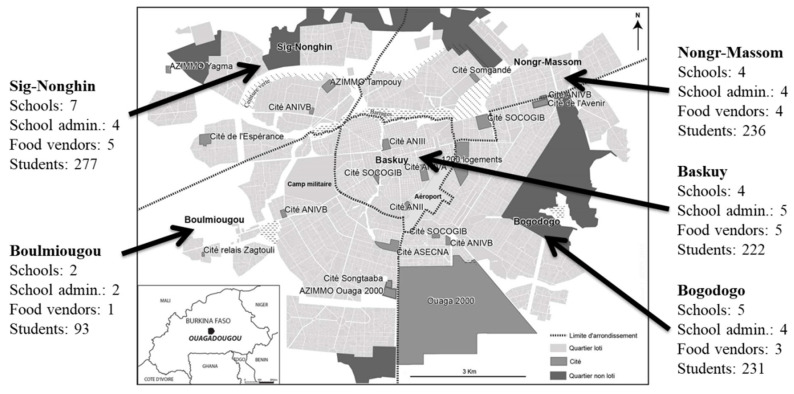
Map of Ouagadougou and the distribution of the three data sources (school administrators, food vendors and in-school adolescents) across the five city districts (map from [20]).

Between March and December 2020, we undertook a cross-sectional study in Ouagadougou, the capital city of Burkina Faso (West Africa). We used the following sampling strategy. First, we obtained a list of all 536 junior secondary schools in Ouagadougou with enrollment numbers for each grade. Junior secondary schools are called “Collège” in Burkina Faso and cover four years of education. The grades are counted in reverse order, wherefore 6 is the lowest and 3 the highest grade in junior secondary schools (equivalent to grades 7 to 10), which are in general visited by adolescents aged 11 to 15 years. Our aim was to recruit 20 schools, 60 students from each school, and 15 students from each grade 6 to 3, resulting in a total sample size of 1200 students. Because of our sampling targets, we dropped all schools that did not have at least 15 students per grade, resulting in 388 available schools from which to sample. We then used stratified random sampling by district to select 5 junior secondary schools each in Baskuy (40 schools on list), Nongr-Massom (46), Bogodogo (100), Boulmiougou (129) and Sig-Nonghin (73) (see Figure 1). We were unable to determine the proportion of girls and boys per class in advance. Therefore, on the day of the school visit, trained field workers were instructed to recruit an equal number of boys and girls (or as many close to equal) per grade using convenience sampling. In addition, field recruiters aimed to recruit 20 school administrators (one from each school) and 20 food vendors (two from 10 randomly selected schools out of the 20).

### 2.2. Data Sources and Study Participants

Data collection was locally managed by the *Institut Superieur des Sciences de la Population* (Higher Institute of Population Sciences, ISSP) in Ouagadougou, Burkina Faso. The field workers were employed and trained by the ISSP, which specializes in social science research and data collection. After the training, the field workers visited all of the sampled schools to collect information from the school administrators, food vendors, and in-school adolescents using structured questionnaires.

(1) *School administrator survey*: From each selected school, the field workers identified school administrators that were knowledgeable of or responsible for food and health environment practices/activities in the school. The survey included questions on existing health-related policies or guidelines, provision of health and nutrition services, the delivery of a nutrition curriculum, provision of WASH facilities and services, physical activity classes, and access to medical screening and mental health services. In case a school administrator reported about the available policy or guideline, we asked if it included a recommended package of school-based health and nutrition services.
(2) *Food vendor survey*: The field workers identified two formal or informal food vendors that were located within or immediately surrounding the property of the included schools. This survey covered several topics including the food items sold by the vendors, their costs and the proportion of sales of these foods.
(3) *Adolescent survey*: The field workers randomly select 60 adolescents per school, with 15 students each from each grade. The questionnaire covered the following topics: demographic characteristics, hygiene practices, physical activity, food preferences and eating behaviors, food security, peer influences, and mental health. The questionnaire was administered by the field workers, who assessed the diet of the adolescents through a 24 h dietary recall based on meal time (breakfast, lunch, dinner and snacks) listing 26 pre-defined food groups. Food groups do not define individual food items (e.g., “other fruits” compared to “bananas”), but provide sufficient detail to understand dietary practices.

### 2.3. Data Analyses

We applied descriptive statistics for the data provided by the school administrators, food vendors and in-school adolescents. We display the data by frequencies and as proportions. We structured the results according to study objectives describing: (1) the availability of health-related policies or guidelines enacted by the schools, (2) the health, nutrition and WASH services provided in schools, and (3) the school food environment including food vendors. These objectives cover broadly three out of the four environment dimensions to assess the school food environment as recommend by O’Halloran et al. [11], which are the physical, economic, socio-cultural and policy environment. We used the school administrator data to assess components 1 and 2 and the food vendor and student data to assess component 3. Data were analyzed in Stata/IC 15.0 (StataCorp, College Station, TX 77845, USA) [19].

## 3. Results

We recruited a total of 22 junior secondary schools for our study, two more than our target of 20 schools. This is because for two of the target schools, we could not recruit the expected 60 students. We therefore randomly selected two additional junior secondary schools from the list as described in Section 2.1. to reach a sample of 1200 student in total. Because data collection was interrupted by the lockdowns due to the COVID-19 pandemic, we could not reach our target of 20 administrators, 1200 students, and 20 food vendors. Our final sample therefore consisted of (i) 19 school administrators, (ii) 18 food vendors located within (15 food vendors) or immediately surrounding the school property (three food vendors), and (iii) 1059 in-school adolescents from 22 schools. Figure 1 displays the distribution of our data sources across the five city districts of Ouagadougou. The adolescents were equally distributed (±22% each) across the five districts with slightly less students (9%) in Boulmiougou.

### 3.1. Component 1: Availability of Health-Related Policies, Guidelines and School Curricula

Questions on the availability of health-related policies or guidelines were answered by the 19 school administrators. Table 1 displays the number of available policies and guidelines at the school or regional level. Accordingly, seven out of the 19 school administrators reported that their school had a health-related policy or guideline and only four of the school administrators were aware of such a policy or guideline at the regional level.

Six school administrators were aware of a recommended package of school-based health and nutrition services at the school level and four school administrators knew about it at the regional level (Table 1). At the school level, all six school administrators reported that this package included food vendor guidelines or regulations. Five out of the six school administrators were aware of it covering physical activity, three of deworming and use of first-aid kits each, and one of WASH infrastructure/practices. With regard to nutrition, only two school administrators reported that it included a school feeding program and only one reported about school gardens. According to the six school administrators, the package of school-based health and nutrition services did not include information on micronutrient supplementation, dietary guidelines, a mandated health/nutrition curriculum, counselling or referral of staff or students on health problems/behaviors or health screening services. At the regional level, all four school administrators, who were aware of such a package, reported that it included information on physical activity, two on WASH infrastructure/practices and one on deworming, school feeding programs, school gardens, and medical check-ups. All other topics were not covered.

Table 2 shows the results for the availability of a health and nutrition curriculum, as reported by the school administrators. Only three out of the 19 school administrators were aware that their school provided such a curriculum. Out of these three school administrators, all of them reported that the curriculum covered healthy eating practices, two mentioned physical activity and reproductive health education each, and one knew about information covering unhealthy foods and beverages, hygiene, preventive health and general life-skills. According to these three school administrators, their schools did not have a curriculum on emotional and mental health or violence prevention.

### 3.2. Component 2: Health, Nutrition and WASH Services Provided in Junior Secondary Schools

As reported by the 19 school administrators, two out of the 19 schools had a designated health and/or nutrition teacher. Three schools had a designated staff member to deliver or provide referrals needed concerning health and nutrition services during the last two years.

Table 3 shows the available school services provided during the same or previous year of the survey and the frequency at which they were provided as reported by the 19 school administrators. These services cover health, nutrition and WASH. With regard to health services, 17 out of the 19 schools provided physical activity education. Out of these schools, 16 provided this service weekly. According to five school administrators, their schools provided deworming services on a daily (two schools), monthly (two schools) or yearly (1 school) basis. Two schools provided daily counseling services, two schools daily infections screening or medical check-ups and one school provided yearly infections screening. None of the schools provided vision, hearing, dental/oral health or other screening services or took height or weight measurements. With regard to nutrition services, only two school administrators were aware that school feeding programs were in place. None of the schools provided micronutrient supplementation.

Various WASH-related services were provided by the schools. Of the 19 schools, 14 provided drinking water during the same or previous year of the survey. The main water source was tap or piped water for 13 out of 19 schools. 17 out of the 19 schools had toilets for the students with 12 schools having gender-specific toilets. Of the 19 schools, 15 had private toilets separated by a door or entryway wall and 13 provided handwashing stations. Five out of the 14 schools cleaned their toilets daily, eight schools weekly and one school monthly. Eleven of the 19 schools also repaired their toilets on a yearly, monthly or daily basis and four schools provided both water and soap in the handwashing stations. Five of the 19 schools had handwashing stations in the canteen or eating area, whereas one had them close to the toilets. Sanitary napkins/pads for girls were offered by only one school and only on a monthly basis.

Additionally, the school administrators answered questions on sports and physical activity. Of the 19 school administrators, 17 reported that their schools provided at least one form of physical activity. Most schools offered sports once to twice per week. As shown in Table 4, the most common sports offered were running (17 schools), soccer (13 schools), and rope skipping (nine schools). Running and soccer were also the most common activities for both genders. Each of these activities took place for a mean of 44 to 62 min per day. Basketball and volleyball were offered by four schools and netball by one school. Swimming classes and tennis were not available at any of the schools. There was no specific physical activity offered for girls only, but three of the 19 schools offered football, netball and volleyball to only the boys. Ten schools also offered sports that were not captured by the survey and, thus, were not further defined.

### 3.3. Component 3: The Food and Health Environment in Junior Secondary Schools

In order to assess the food and health environment, the school administrators were asked to report on the available school facilities (Table 5). Accordingly, 12 schools provided access to a playground or track field. Only one school offered changing rooms for girls only. A school clinic was integrated into three schools. With regard to the food environment, six schools each had a kitchen or a food storage facility. A cafeteria or canteen was accessible in only three schools and a school garden in only two schools.

**Figure 2:**
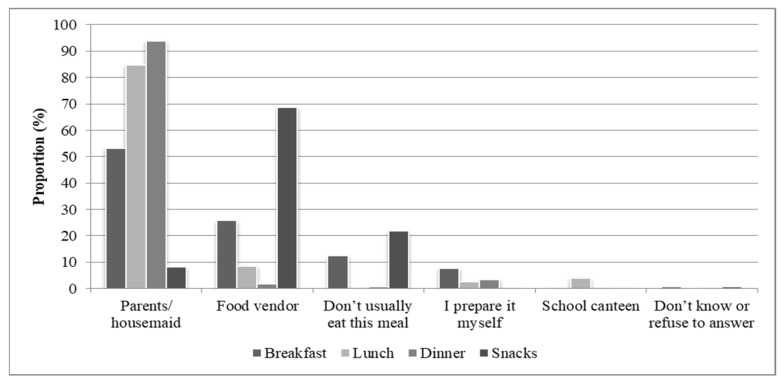
Proportion of meals prepared by source as reported by 1059 students from the 22 junior secondary schools in Ouagadougou.

**Figure 3:**
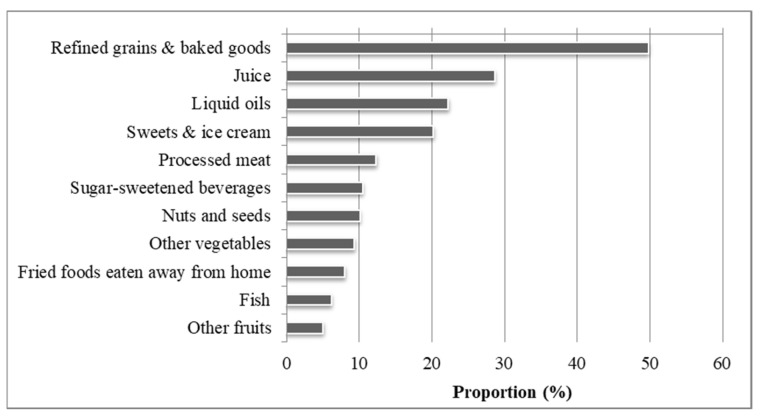
Snacks purchased by 534 out of 1059 in-school adolescents over the previous 24 h. Note: Snacks consumed by <5% of the students are listed here: low fat dairy, red meat, deep orange fruits, high fat dairy, whole grains, dark green leafy vegetables, white roots and tubers, legumes, deep orange vegetables, citrus fruits, hot beverages, eggs, deep orange tubers, poultry, and cruciferous vegetables.

When the in-school students (*n* = 1059) were asked who prepared their meal during the day, only 4% reported going to the school canteen for lunch and none went there for breakfast, dinner or snacks (Figure 2). The majority of the students received their meals from their parents or housekeeper: 53% for breakfast, 85% for lunch, and 94% for dinner. Less than 10% of the students reported that they prepared the meals themselves and 12% reported that they did not have breakfast in the morning. With regard to snacking, 78% of the students (*n* = 799) consumed snacks, which were purchased by 69% of these students from food vendors located within or immediately surrounding the school property. Specifically, 26% purchased their breakfast and 8% their lunch from a food vendor. Of those students who purchased snacks the previous day (*n* = 534), the majority consumed refined grains and baked goods, juices, liquid oils (e.g., olive oil, vegetable oil, or coconut oil), sweets and ice cream, processed meat, and sugar-sweetened beverages (Figure 3).

Table 6 shows the food items that were sold by the food vendors on the day of the survey as reported by 18 food vendors located in or around the junior secondary schools from this study. The majority of foods sold were snacks, processed foods and beverages. Accordingly, 14 food vendors sold snacks, 13 processed foods and 10 beverages. The snacks included among others peanuts, sweet sesame, dried fruits and biscuits, the processed foods covered sandwiches filled with meat, fish, soya or avocados, and the beverages comprised water, sweet beverages and fruit juices. Deep fried foods such as fried fish and bananas were only sold by four food vendors and dairy products by one food vendor. Fresh fruits were sold by three food vendors and were also the cheapest items with a mean price of 0.05 USD per fruit. Beverages were the most expensive items sold with a mean price of 0.38 USD per bottle.

## 4. Discussion

In this cross-sectional study in Ouagadougou, Burkina Faso, we describe the food and health environment of junior secondary schools based on information provided by 19 school administrators, 18 food vendors, and 1059 in-school adolescents. Motivated by the WHO NFSI framework, we focused our evaluation on three components: (1) the availability of health-related policies or implemented guidelines, (2) the provision of health, nutrition and WASH services, and (3) the quality of the school food environment, including foods sold by vendors.

We found that the junior secondary schools lacked health-related policies or guidelines (component 1). Of the 19 school administrators interviewed, only seven were aware of a health-related policy or guideline in place and of those only six included a recommended package of school-based health and nutrition services. In addition, only three school administrators were aware of an available school health and nutrition curriculum. We found that the provision of health, nutrition and WASH services was low (component 2). While 17 out of 19 schools provided a physical activity curriculum, there was insufficient provision of WASH services, medical check-ups, disease prevention screening, micronutrient supplementation, or sanitary facilities. We also report that the school food environment, which included foods sold by vendors (component 3), was inadequate. Only three out of 19 schools had school canteens, which may explain why most students obtained their meals from their parents or the housemaid. Of the whole sample of students (N = 1059), 26% consumed their breakfast and 8% their lunch from a food vendor located within or immediately surrounding the school property. The food vendors provided mainly snacks and processed foods, with only three schools offering fresh fruits such as bananas, mangos, and oranges.

In the next sections, we discuss the implications of our findings and provide some lessons learned to inform policy and improve the school food and health environment across schools in Ouagadougou.

### 4.1. Study Implications and Lessons Learned

#### 4.1.1. Promote the Implementation of School Food and Health Policies and Guidelines

In 2004, the World Health Assembly endorsed the “WHO Global Strategy on Diet, Physical Activity and Health” (DPAS) due to an increase in non-communicable diseases (NCDs) and to counteract its major risk factors. This strategy encourages governments to develop and implement “school policies and programs that promote healthy diets and increase levels of physical activity” [21]. The DPAS School Policy Framework builds on existing WHO initiatives (of which the NFSI is one) and aims to guide policy-makers in the development and implementation of these policies [21]. However, research has shown that most schools have failed to implement these nutrition policies at the school level [11]. In our study, the majority of the interviewed school administrators were not aware of national or regional health policies for schools. Further, only seven out of the 19 school administrators knew that a health policy was implemented at their school.

The WHO policy framework recommends implementing a school curriculum on healthy foods and beverages, a food service environment that includes quality standards, an environment for physical activity, and the promotion of health services by school staff [21]. Although six of the seven schools included health and nutrition services, these were inadequate and did not include basic services such as a school feeding program, WASH infrastructure, dietary guidelines, medical check-ups, or screening services. Further, only six of the school administrators were aware of food vendor guidelines. Food vendor guidelines address the kind of foods that should be sold as well as the hygiene conditions. The WHO also encourages governments to promote the use of nutritional standards by food vendors who are present on or near the school premises [21].

Studies have found that school food and nutrition policies can change the school food environment and influence better food choices for the entire student population, as opposed to only an individual [22]. A systematic review and meta-analysis on the effectiveness of school food environment policies identified that meal standardization, which included fruits and vegetables, increased their consumption and improved the dietary behaviors of school children. However, evidence is still scarce as the majority of studies were conducted in industrialized countries [6]. In one study from Burkina Faso, no schools had a written nutrition policy in place and access to clean water, or hygienic food, and toilet facilities were inadequate in three out of the six schools [17,23]. A study conducted in South Africa found that after the implementation of school policies various school-based interventions were put in place such as increasing the availability of healthier foods at school canteens or among food vendors or such as providing nutrition education to the students [24]. These interventions were identified to have contributed to at least minor improvements in health outcomes. Similar findings were made in Mexico [25] and Brazil [26]. Without policies in place, the implementation of various nutrition interventions is a challenge. The integration of such policies should be promoted and guaranteed in the school environment.

#### 4.1.2. Improve the Availability of Health, Nutrition and WASH Services in Schools

Based on our study findings, we encourage to improve the school environment as set out in the WHO NFSI [16]. One potential intervention point is through qualified school teachers. Well-trained teachers can change adolescents’ food choices and attitudes by providing nutrition education and sharing knowledge about healthy eating and practices [27,28]. However, one challenge is that the proportion of qualified teachers in sub-Saharan Africa is low, representing 64% of primary and 50% of secondary school teachers. This proportion has constantly declined since 2000 as schools increasingly hire less qualified contract teachers at lower costs [8]. Out of 19 schools in our study, only two had a designated health and/or nutrition teacher and three schools had a designated staff member to deliver health and nutrition services over the last two years. In addition, the majority of schools did not have a health and nutrition curriculum. Hence, 16 out of 19 schools did not provide lessons on healthy eating and 18 schools did not provide lessons on unhealthy foods or beverages.

A study similar to ours was conducted among 16 secondary schools in South Africa to evaluate the food and nutrition environment linked to government policy programs, school health packages and services, which included health assessment and screening, health and nutrition education and promotion and on-site services by school nurses, and foods sold [29]. The authors reported that the South African government introduced an Integrated School Health Program (ISHP) in 2012 to improve the general health of in-school adolescence and promote their food and nutrition environment. Most of the teachers were trained by the Department of Education, others through workshops, self-study or as part of the teaching training. The curriculum included teaching on healthy eating, a balanced diet, ill health, harmful substances in food production and physical education/exercise on a weekly or bi-weekly basis [29]. Despite this initiative, they found not only a lack of designated school staff, but also that the majority of teachers who taught nutrition had no formal nutrition training and showed poor knowledge on questions related to healthy diets. Thus, even though nutrition was included in the curriculum and considered a success of the ISHP, the quality of nutrition training and knowledge on healthy diets of school administrators also in Burkina Faso should receive closer attention and further investigation.

Our findings show that there was an inadequate level of health services provided by the schools. Specifically, there was no provision of micronutrient supplementation and screening services for vision, hearing, and oral health. Only two of the 19 schools screened for common illnesses or infections. The implementation of WASH practices was low, with under one-quarter of schools providing soap and water at the handwashing stations. Numerous health benefits have been associated with cleaning hands with both soap and water, which can reduce one-third of diarrhea cases among school children [30]. Further, only 5% of schools had gender-specific dressing rooms and just under two-thirds of schools had gender-separated toilets. A lack of (unisex) toilets was also recognized in a study conducted in rural Burkina Faso [10].

In this study, we found that involvement in physical activity in schools was high and common sports such as running and soccer were provided. However, only eight out of 19 schools had a playground or track-field. Further, three of the schools offered common sports such as football, netball, and volleyball to only the boys. These results suggest that more needs to be done to address the low participation of girls in school-based physical activities. The WHO recommends that children and adolescents should be involved in at least 60 min of physical activity daily for optimum health outcomes [31]. Research has shown that low physical activity in early adolescence carries major health implications into adulthood [32]. The availability of sports facilities can increase student participation in physical activities and school lessons [33].

#### 4.1.3. Strengthen the School Food Environment

We suggest that more progress needs to be made to strengthen the availability of nutritious foods in the school environment in Burkina Faso. These findings were also emphasized by Pacific et al. [18], who conducted a systematic literature review on the contribution of home and school environment on children’s weight and food choices in Africa. They found ten Africa-based studies, which confirmed that the perception and availability of healthy foods provided at home and in schools positively impacted the children’s food choices [18], while equally healthy foods provided by school food vendors may decrease the odds of obesity among the schoolchildren [34]. Subsequently, an integrated approach should be applied. We recommend strengthening the school food environment through the provision of school feeding programs, food vendor regulations and the integration of gardens at the school and community level.

Our study revealed that approximately only one-quarter of schools had a school feeding program in place. School feeding programs can alleviate hunger and reduce micronutrient deficiency, anemia and obesity, and may equally positively impact school attendance, academic performance and gender equality [35,36,37]. In 2018, a presidential initiative was launched to strengthen school feeding programs in Burkina Faso through domestic financing and a multi-sectoral approach [38]. School feeding programs such as those initiated by the FAO focus on supporting the infrastructure and capacity for food provision to increase the availability and diversity of foods provided in schools [39]. In the schools of our study, only six out of 19 schools had a kitchen or food storage and three schools had a designated cafeteria or canteen. Likely because of the lack of school canteens, students have to look for other food sources from food vendors or meals provided by their parents. In order to make good and healthy food choices, schools should encourage teachers and school health nurses to integrate healthy eating into their curriculum and provide nutrition guidelines or even regulations for food vendors.

A study by Okeyo et al. [29] among 16 secondary schools in South Africa also assessed the food environment through food vendors. Accordingly, most of the South African students (59%) bought their food from vendors. The main food items purchased by the students were unhealthy energy-dense foods such as chips, cakes/donuts, and chocolates. Fruits were bought by only 11% of the students. Our survey confirmed these findings as food vendors mostly sold unhealthy snacks and processed foods when compared to fruits, which were the least sold (14 versus three out of 18 food vendors) and consumed by the in-school adolescents. Food vendors play a critical role in the school environment, as 69% of students in our study bought snacks from food vendors. It may be convenient for students to buy snacks from food vendors especially in schools without a formal feeding program; however, they can be unhealthy and high in fat and sugar [40,41]. Hence, food vendors should be encouraged to sell fruits and vegetables as snacks rather than energy-dense alternatives [42]. Studies in Burkina Faso and El Salvador were able to address this issue by training the vendors in healthy preparation of their foods and convinced some of them of the benefits of selling healthier drinks and fruits to students [17,43].

Our results also show that most schools (90%) did not cultivate their own vegetable gardens. School gardens may allow students to learn about healthy eating and the production of fresh vegetables [29]. Previous research has reported that increased fruit and vegetable consumption is positively associated with academic performance [44] and reduces the risk for micronutrient deficiencies and non-communicable diseases [45]. Further, a Burkina Faso study found that community engagement played an important role in the promotion of school vegetable gardens as both community members and parents helped with preparing the land and finding water for the gardens, especially during the dry season [46]. The authors of the study reported that successful community engagement in school garden projects significantly increased the student’s knowledge of good nutrition intake [46]. However, school gardens also have their challenges such as due to their seasonality, required time commitment, and small output [29]. An alternative may be the enhancement of community or home gardens as over half of the students in our study received their meals from their parents. Parents were found to act as role models, shape positive behavior change and modify available foods at home [47]. Campaigns should therefore also target the parents and community through the promotion of healthy eating behavior and attitudes [16,18].

Overall, our study is in line with other research on school-based programs, which continue to reveal many challenges for implementing school-based interventions, including non-existing school nutrition policies, unregulated food environments, inadequate sanitary facilities, and limited financial resources [6,29,36,48].

### 4.2. Study Strengths and Limitations

The study is unique in its current form as we included three sources of information to describe and understand the school food and health environment in junior secondary schools across Ouagadougou: school administrators, food vendors and in-school adolescents. The three data sources complemented each other rather than allowed for triangulation. In this regard, we recognize several study limitations.

First, we did not assess the whole (physical) school food and health environment, but included only selected data sources. Subsequently, we recognize that: (i) only one school administrator per school was interviewed, that (ii) the selection of food vendors might have been biased as not all food vendors in or around the school premises were targeted, (iii) that we excluded outside-school adolescents, who have different health risks than their peers in school [4] and (iv) that we focused only on urban schools and did not differentiate between public and private junior secondary schools.

Second, there exist no standardized food environment instruments and indicators to assess food environments across diverse settings [14]. Overall, the school food environment may be assessed through quantitative methods (e.g., menu analysis, direct observations, questionnaires and surveys), qualitative methods (e.g., semi-structured interviews, focus-group discussions) as well as mixed methods [11]. A review on measurement methods to assess the school food environment including strengths and limitations can be found in O’Halloran et al. [11]. The authors conclude that “there is a need for further research into appropriate measurement methods which are high quality and can be applied broadly across a range of country contexts” [11].

Third, we recognize that we collected self-reported information. Subsequently, we cannot exclude an information bias in favor of desirability rather than reality and/or a misunderstanding or no understanding of questions (e.g., such as on policies and guidelines or the provision of drinking water, which two schools reported to provide “yearly”).

Fourth, this cross-sectional study does not allow for long term assessments and observation of changes over time and their impact on diets, nutritional status or health [14].

Lastly, our study was interrupted by the COVID-19 pandemic in Burkina Faso in 2020. We are unable to determine how this interruption might have affected our results.

## 5. Conclusions

Our study identified a critical need to improve the food and health environment of junior secondary schools in urban Burkina Faso. We identified that there is low awareness on health-related policies and guidelines enacted by the schools, that health, nutrition and WASH services and their integration in the school curriculum are scarce in a majority of schools, that the integration of canteens in schools needs to be promoted, and that food vendors should be regulated to provide healthy eating options to in-school adolescents. School-based interventions are likely to improve not only the health of the early adolescents, but also increase the school enrolment rate in Burkina Faso. We encourage the promotion, improvement and strengthening of the school food and health environment and emphasize further evaluation of its impact on early adolescents’ diet and health.

## Figures and Tables

**Table 1:** Availability of health-related policies or guidelines as reported by the school administrators from 19 junior secondary schools in Ouagadougou.

Health-Related Policies or Guidelines	At the School Level		At the Regional Level	
	*n*	%	*n*	%
Available health-related policies or guidelines	7	37	4	21
Included a recommended package of school-based health and nutrition services	6	32	4	21
**Topics covered by the package:**				
Food vendor guidelines/regulations	6	100	0	0
Physical activities	5	83	4	100
Deworming	3	50	1	25
Use of first-aid kit	3	50	0	0
School feeding program	2	33	1	25
School garden	1	17	1	25
WASH infrastructure/practices	1	17	2	50
Micronutrient supplementation	0	0	0	0
Dietary guidelines	0	0	0	0
Mandated health/nutrition curriculum	0	0	0	0
Counselling or referral of staff or students on health problems/behaviors, incl. mental health	0	0	0	0
Screening services: vision, hearing, height and weight measurement	0	0	0	0
Medical check-up	NA	NA	1	25

Note: NA = Not applicable, question not asked.

**Table 2:** Availability of school health and nutrition curricula as reported by the school administrators from 19 junior secondary schools in Ouagadougou.

School Health and Nutrition Curriculum	*n*	%
Number of schools with a specific school health and nutrition curriculum available	3	16
**Topics covered by the curriculum:**		
Healthy eating practices	3	100
Physical activity	2	67
Reproductive health/sexuality education	2	67
Unhealthy foods and beverages	1	33
Hygiene (including oral)	1	33
Health care-seeking/disease prevention	1	33
General life skills	1	33
Emotional and mental health	0	0
Violence prevention (e.g., bullying, fighting)	0	0

**Table 3:** Provision of health, nutrition and WASH services and their frequency of provision during the same or previous year of the survey as reported by 19 school administrators in junior secondary schools in Ouagadougou.

Health, Nutrition and WASH Services in Schools	Services Provided		Frequency of Service Provision			
			Daily	Weekly	Monthly	Yearly
	*n*	%	%	%	%	%
**Health services**						
Physical activities	17	89	6	94	0	0
Deworming	5	26	40	0	40	20
Counseling services	2	11	100	0	0	0
Common illnesses/infections screening	2	11	50	0	0	50
Medical check-up	1	5	100	0	0	0
Other screening	0	0	0	0	0	0
Vision screening	0	0	0	0	0	0
Hearing screening	0	0	0	0	0	0
Dental/oral health screening	0	0	0	0	0	0
Height and weight measurements	0	0	0	0	0	0
**Nutrition services**						
School feeding programs	2	11	50	0	50	0
Micronutrient supplementation for girls only	0	0	0	0	0	0
Micronutrient supplementation for boys and girls	0	0	0	0	0	0
**WASH services**						
Drinking water	14	74	79	7	0	14
Toilets cleaned	14	74	36	57	7	0
Toilets repaired	11	58	9	0	27	64
Girls sanitary napkins/pads	1	5	0	0	100	0

**Table 4:** Offered sports and their duration per day in junior secondary schools as reported by the school administrators for 19 junior secondary schools.

Sports at School	Sports Offered		Duration per Day (min)		
	*n*	%	Mean	Min	Max
Running (marathon, sprinting)	17	89	46.4	1	120
Soccer	13	68	61.5	20	120
Rope skipping	9	47	44	1	90
Basketball	4	21	60	60	60
Volleyball	4	21	52.5	45	60
Netball	1	5	60	60	60
Swimming	0	0	0	0	0
Tennis	0	0	0	0	0
Other sports	10	53	NA	NA	NA

Note: NA = Not applicable, question not asked.

**Table 5:** Available school facilities as reported by the school administrators for *19 junior secondary* schools.

School Facilities	*n*	%
Toilets	17	89
Handwashing stations	13	68
Toilets by gender	12	63
Playground/track fields	8	42
Kitchen	6	32
Food storage facility	6	32
School clinic	3	16
Cafeteria or canteen	3	16
School garden	2	11
Changing rooms for girls	1	5

**Table 6:** Food items sold on the day of the survey and their mean price as reported by 18 food vendors located in and around junior secondary schools in Ouagadougou.

Foods	Food Items	Number of Food Vendors		Mean Price per Food Item	
		*n*	%	CFA Franc	USD
Snacks	Peanuts, sweet sesame, dried bananas or fruits, milk bonbons, chips, croquette, monkey bread, sandwiches, cakes, popcorn, biscuits	14	78	113	0.21
Processed foods	Meat, fish, soya and avocado sandwiches, sausages, cakes	13	72	120	0.22
Beverages	Water, sweet beverages, fruit juices, local juices, milk	10	56	200	0.38
Deep fried foods	Fried fish and bananas, donuts	4	22	50	0.09
Fresh fruits	Mangos, bananas, oranges	3	17	25	0.05
Dairy products	Yoghurt, eggs	1	6	100	0.19

Note: CFA franc = Franc de la Communauté Financière en Afrique/West African currency; USD = United States Dollar; exchange rate from (01.04.2021) with 100 CFA franc = 0.16 USD.

## Data Availability

The data is archived in the ARISE Network data repository. The data is available upon request from the ARISE data manager at Harvard University.
